# Breast cancer proteomics reveals correlation between estrogen receptor status and differential phosphorylation of PGRMC1

**DOI:** 10.1186/bcr2155

**Published:** 2008-10-15

**Authors:** Hans Neubauer, Susan E Clare, Wojciech Wozny, Gerhard P Schwall, Slobodan Poznanović, Werner Stegmann, Ulrich Vogel, Karl Sotlar, Diethelm Wallwiener, Raffael Kurek, Tanja Fehm, Michael A Cahill

**Affiliations:** 1 Department of Obstetrics and Gynecology, University of Tuebingen, Calwerstraße, 72076 Tübingen, Germany; 2 Current Address: Department of Surgery, Indiana University School of Medicine, W Walnut Street, Indianapolis, Indiana, 46202, USA; 3 ProteoSys AG, Carl-Zeiss-Straße, 55129 Mainz, Germany; 4 Department of Pathology, University of Tuebingen, Liebermeisterstraße, 72076 Tübingen, Germany; 5 Current Address: Department of Pathology, Ludwig-Maximilians-University of Munich, Thalkirchnerstraße, 80337 Munich, Gemany; 6 Current Address: Merck-Serono – Global Clinical Development Unit Oncology, Merck KGaA, Frankfurter Straße, 64293 Darmstadt, Germany; 7 School of Biomedical Sciences, Charles Sturt University, Wagga Wagga, NSW, 2678, Australia

## Abstract

**Introduction:**

Breast tumors lacking the estrogen receptor-α (ER-α) have increased incidence of resistance to therapy and poorer clinical prognosis.

**Methods:**

Whole tissue sections from 16 cryopreserved breast cancer tumors that were either positive or negative for the ER (eight ER positive and eight ER negative) were differentially analyzed by multiplex imaging of two-dimensional PAGE gels using 54 cm isoelectric focusing. Differentially detected spots of Progesterone Receptor Membrane Component 1 (PGRMC1) were shown to differ in phosphorylation status by differential two dimensional polyacrylamide gel electrophoresis of phosphatase-treated tumor proteins. Site directed mutagenesis was used to create putative phosphorylation site point mutants in PGRMC1. Stable transfectants of these mutants in MCF7 cells were assayed for their survival after oxidative stress, and for AKT kinase phosphorylation. Immune fluorescence using anti-PGRMC1 monoclonal antibody 5G7 was performed on breast cancer tissue microarrays.

**Results:**

Proteins significantly differentially abundant between estrogen receptor negative and estrogen receptor positive tumors at the 0.1% level were consistent with published profiles, suggesting an altered keratin pool, and increased inflammation and wound responses in estrogen receptor negative tumors. Two of three spots of PGRMC1 were more abundant in estrogen receptor negative tumors. Phosphatase treatment of breast tumor proteins indicated that the PGRMC1 isoforms differed in their phosphorylation status. Simultaneous mutation of PGRMC1 serine-56 and serine-181 fully abrogated the sensitivity of stably transfected MCF7 breast cancer cells to peroxide-induced cell death. Immune fluorescence revealed that PGRMC1 was primarily expressed in ER-negative basal epithelial cells of mammary ductules. Even in advanced tumors, high levels of ER or PGRMC1 were almost mutually exclusive in individual cells. In five out of five examined ductal *in situ *breast cancers of comedo type, PGRMC1 was expressed in glucose transporter 1 negative or positive poorly oxygenated cells surrounding the necrotic core, surrounded by a more distal halo of ER-positive cells.

**Conclusions:**

PGRMC1 phosphorylation may be involved in the clinical differences that underpin breast tumors of differing ER status.

## Introduction

Breast cancer is among the most common forms of cancer observed in women, with approximately 185,000 new cases and 40,000 deaths estimated in the USA in 2008 [1]. Endogenous estrogens, which have effects on many organs, are thought to play a major role in the development of the breast, suggesting that increased sensitivity or longer exposures to estrogens is involved in greater risk for tumorigenesis [2-4].

The classical estrogen receptor (ER)-α is found in 50% to 80% of breast tumors and ER-α status is essential in making clinical decisions about endocrine therapy with anti-estrogens, which inhibit the mitogenic activity of estrogens in breast cancer. There are three classes of anti-estrogens currently in clinical use: selective estrogen receptor modulators (for example, tamoxifen); aromatase inhibitors; and 'pure' estrogen antagonists such as fulvestrant, which – like tamoxifen – binds to ERs competitively. However, in contrast to tamoxifen, fulvestrant's binding leads to rapid degradation and loss of the ER-α protein [5,6].

Clinically, a positive ER-α status correlates with favorable prognostic features, including a lower rate of cell proliferation and histologic evidence of tumor differentiation. ER-α status is also prognostic for the site of gross metastatic spread. For reasons unknown, ER-α-positive tumors are more likely to initially manifest clinically apparent metastases in bone, soft tissue, or the reproductive and genital tracts, whereas ER-α-negative tumors more commonly metastasize to brain and liver. Several studies have correlated ER-α expression with lower Matrigel invasiveness and reduced metastatic potential of breast cancer cell lines [7,8]. Moreover, when ER-α-positive cells are implanted in nude mice, tumors appear only in the presence of estrogens and are poorly metastatic as compared with those developed from ER-α-negative breast cancer cell lines [9,10]. This paradox suggests that ER-α expression could be associated with or involved in pathways that hinder cancer progression.

At the transcriptome level, gene expression analysis has revealed that different molecular subtypes exist within ER-α-positive and ER-α-negative breast cancers, and these are associated with different clinical outcomes. ER-α-positive tumors exist in at least two subtypes, luminal A and luminal B, which vary markedly in terms of gene expression and prognosis [11]. Conversely, hormone-receptor-negative breast cancer comprises two distinct subtypes, the Her2 (human epidermal growth factor receptor 2) subtype and the basal-like subtype [11,12], which differ in biology and behavior, and are both associated with a poor outcome.

Importantly, a very similar subdivision of breast cancers has been produced based upon immunohistochemistry, conducted to analyze patterns of protein expression in tumor sections, which suggests that a few protein biomarkers can be used to stratify breast cancers into different fundamental groups [13,14]. One set of biomarkers comprises the family of cytokeratins (CKs). They can be grouped into the 'luminal CKs' (CK-7/8, CK-18, and CK-19) and into the 'basal CKs' (CK-5/6 and CK-14) [13].

In addition to these molecular portraits, it has been shown that expression patterns present in primary breast cancers are also observed in their respective metastases [15]. Other gene expression profiles have distinguished breast cancers according to the differential expression of a wound response signature. More than 20 years ago, based on histologic similarities between tumors and wound healing, Dvorak [16] proposed that the tumor stroma is 'normal wound healing gone awry'. Since then it has been discovered that genes induced in a fibroblast serum-response program are expressed in tumors by the tumor cells themselves, by tumor-associated fibroblasts, or both [17]. The molecular features that define this wound-like phenotype are evident at an early clinical stage, persist during treatment, and predict increased risk for metastasis and death in breast, lung, and gastric carcinomas.

We previously published a system for proteomic analysis involving differential radioactive labeling of samples and separation using 54 cm immobilized pH gradient (IPG)-isoelectric focusing (IEF) [18,19]. In the present study we used this system to identify protein species with pronounced and consistent differential abundance between sample categories. We employed large homogenous invasive ductal breast carcinomas, which are well suited for conventional proteomics analysis, but are becoming increasingly rare because of improved screening programs. Differential proteomic analysis of pooled tumors that were selected on the basis of being either ER-α-positive or ER-α-negative unexpectedly revealed differentially abundant phosphorylated isoforms of the cytochrome b5-domain protein progesterone receptor membrane component (PGRMC)1 (reviewed in [20]) between these tumors.

## Materials and methods

### Quantitative population multiplex proteomics

The tumors used in the present study were from a tissue archive previously described [19]. Tumors with large homogenous lesion regions were selected, assayed for RNA integrity, and classified as being either positive or negative for ER-α, as described previously [19]. Frozen tumor sections of 10 μm were lysed directly into SDS buffer, separately iodinated in inverse replicates with each of 125I and 131I, and separated by 54 cm daisy chain IPG-IEF after sample pooling, as described previously [18]. Radioimaging, image processing with the Pic/GREG software (Fraunhofer Institute for Applied Information Technology, 53754 Sankt Augustin, Germany), statistical analysis of the gels, and identification of proteins by mass spectrometry were performed as described previously [19]. There were no identifications of multiple proteins from single spots. The peptide mass fingerprinting (PMF) MASCOT scores [21] of all identifications are provided, indicating the reliability of individual probability-based protein identification assignments.

### Shrimp alkaline phosphatase analysis

Cryogenic slices from six patients (30 slices T433, 40 slices T443, 40 slices T469, 40 slices T470, 35 slices T623, and 30 slices T640) were each extracted with 200 μl aliquots of shrimp alkaline phosphatase (SAP)-dephosphorylation buffer (50 mmol/l Tris [pH 8.5], 5 mmol/l MgCl_2_, 0.25% 3-[(3-cholamidopropyl)dimethylammonio]-1-propanesulfonate, supplemented with 1× EDTA-free complete protease inhibitor cocktail [F. Hoffmann-La Roche Ltd., Basel, Switzerland]). This precooled buffer was added directly on ice to the frozen slices in Eppendorf tubes and the tissue was mechanically homogenized using a plastic pellet pestle. Tubes were vortexed and incubated for 30 minutes at 4°C, followed by centrifugation for 15 minutes at 14,000 *g *at 4°C. Supernatants were collected and pooled together, and the protein concentration was assayed using the BCA method, as described previously [19]. The yield was approximately 4 mg protein. Thirty units of SAP in 30 μl were added into 800 μg of protein in 400 μl in SAP-dephosphorylation buffer, followed by mixing and incubation for 16 hours at 37°C. In parallel, a mock incubation control was performed on 800 μg protein in the same buffer without addition of SAP and containing the following phosphatase inhibitors: activated vanadate, sodium fluoride, and sodium glycerophosphate at final concentrations of 1 mmol/l, 5 mmol/l, and 5 mmol/l, respectively. The incubation was performed in parallel at 37°C for 16 hours. After incubation the proteins were frozen at -80°C. A nonincubated raw lysate control containing 800 μg protein in 400 μl SAP buffer was frozen at -80°C without additions or incubation. Frozen protein mixtures were thawed, precipitated, and resuspended at 1 μg/μl in boiling 0.1 M Tris, 2% SDS (pH8.5). Sixty micrograms of protein were then used for iodination with each of 125I or 131I. Differential inverse replicate ProteoTope analysis was as described above for 54 cm daisy chain IPG-IEF after rehydration loading overnight to pH 5 to 6 IPG (ProteoSys AG, Mainz, Germany).

### Cell culture

MCF-7 cells stably transfected with PGRMC1 and mutants, respectively, were established. Five micrograms of expression plasmid pcDNA3.1 containing hemaglutinin (HA)-tagged mPR (PGRMC1) wild-type or HA-tagged mutants S56A, S180A, S56A/S180A, S56A/C128S/S180A, Y138F, Y179F, or Y179F/S180A were transfected into MCF-7 breast cancer cells. For transfections, a transfection device and kits from AMAXA Biosystems (Gaithersburg, MD, USA) were used, in accordance with the manufacturer's recommendation. A total of 2 × 10^6 ^cells were transfected with circular plasmids and plated with Roswell Park Memorial Institute (RPMI)-medium for 24 hours. Then medium was changed to RPMI medium complete medium containing 60 μg/ml hygromycin B and cells were cultured for 2 weeks for selection of stable integration events. After 2 weeks single colonies had formed and limiting dilution assays were performed to select for colonies grown from a single cell. Colonies were trypsinized, counted, and diluted in twofold dilutions to obtain clones.

For functional assays MCF-7 cells and derivatives were maintained in RPMI medium containing 10% fetal calf serum (FCS), 2 mmol/l L-glutamine, and penicillin and streptomycin. PGRMC1 expression in all cell lines was confirmed by Western blot using an HA-specific monoclonal antibody [22]. Charcoal/dextran-treated FCS was obtained from Hyclone (Acros Organics N.V., Geel, 2440 Belgium; Catalogue Nr SH30068).

### Cell viability assays

Cell viability was measured using an assay that utilized the fluorometric determination of ATP levels as an indicator of cell viability, namely the ATP-TCA kit (TCA-100; DCS Innovative Diagnostik Systeme, Hamburg, Germany), as follows. Cells were incubated with 50 μl tumor cell extraction reagent and 50 μl RPMI medium for 30 minutes at room temperature. Then, 50 μl supernatant was transferred into a 96-well plate and 50 μl chemoluminescence reagent was added. Chemoluminescence was quantified using a luminescence reader (Berthold, Bad Wildbad, Germany). The fluorometric readout is presented in relative light units (higher relative light unit values are associated with higher ATP levels and therefore higher levels of cell viability).

### Phosphorylation of Akt

A total of 1 × 10^6 ^cells were seeded in a six-well plate and cultured overnight at 37°C and 5% carbon dioxide in RPMI medium and 10% FCS. Cells were treated with 1 mmol/l H_2_O_2 _in RPMI for 30 minutes at 37°C and 5% carbon dioxide. Subsequently, cell culture dishes were transferred immediately to ice and lysed in M-PER mammalian protein extraction reagent containing protease inhibitors. Protein concentration was determined using the BCA Protein Assay Kit (both from Pierce, Rockford, IL, USA). Twenty micrograms of protein was loaded per lane onto a 10% polyacrylamide gel and separated by electrophoresis.

The gel was blotted on Hybond ECL nitrocellulose membrane (Amersham, Piscataway, NJ, USA) and blocked overnight at 4°C using 5% milk in TBST buffer. Western blot for P-Akt was performed using rabbit anti-P-Akt 1/2/3 (1:200; Santa Cruz Biotechnology, Santa Cruz, CA, USA) for 1 hour at room temperature. The following antibodies were incubated with a single membrane: mouse anti-Akt-1 to detect total Akt (1:200; Santa Cruz Biotechnology), rabbit anti-HA-probe to detect HA-tagged exogenous PGRMC1 (Y-11; 1:200; Santa Cruz Biotechnology), and rabbit anti-actin (I-19; 1:500; Santa Cruz Biotechnology). As secondary antibodies, either goat anti-rabbit IgG-horse raddish peroxidase (1:2,500; Santa Cruz Biotechnology) or biotinylated anti-mouse IgG (H+L; 1:2,000; Vector laboratories, Burlingame, CA, USA) followed by streptavidin/HRP (1:1,000; DakoCytomation, Hamburg, Germany) were applied. Chemoluminescence was generated using ECL Western Blotting Analysis System (Amersham). The signals were measured with a Lumi-Imager (Boehringer, Mannheim, Germany).

### Immune fluorescence analysis

Monoclonal anti-PGRMC1 antibody 5G7 was generated in mice inoculated with the cytoplasmic domain (Δ43hpr6) of bacterially expressed PGRMC1, representing a 25 kDa protein of which the amino-terminal 43 amino acids of hpr6 were deleted. For immune fluorescence analysis, paraffin-embedded breast cancer tissue was used. Paraffin-embedded sections (5 μm) of a tissue microarray were prepared and labeled with antibodies, in accordance with standard protocols. Sections were deparaffinized and boiled in 10 mmol/l citrate buffer for 4 minutes in a pressure cooker to expose the antigen. Blocking of unspecific antibody binding sites was performed with goat normal serum (10% dilution in antibody diluent [DakoCytomation; Dako Denmark A/S, 2600 Glostrup, Denmark]). Slides were incubated for 60 minutes with the first antibody and rinsed in three changes of phosphate-buffered saline for 1 minute each. The secondary antibody was then applied to the slide for 60 minutes. Sections were washed again to remove the unbound antibody. For counter staining, Vectashield (Vector Laboratories) was used to embed labeled tissue. Labeling reactions were performed in a humified chamber. The following primary antibodies were used: 5G7 monoclonal antibody (1:10), ER-α (1: 200; DCS, Hamburg, Germany) and glucose transporter (GLUT)1 (1:100; Dianova, Hamburg, Germany). Goat anti-mouse AlexaFluor 594 and goat anti-rabbit AlexaFluor488 (both 1:1,000; Invitrogen, Karlsruhe, Germany) were used as secondary antibodies. Preincubation of the 5G7 with recombinant PGRMC1 protein was performed overnight at 4°C at a protein concentration of 0.5 μg/ml. Control was treated in the same way but without recombinant protein.

## Results

### Quantitative multiplex proteomic analysis of pooled tumor samples

**Figure 1:**
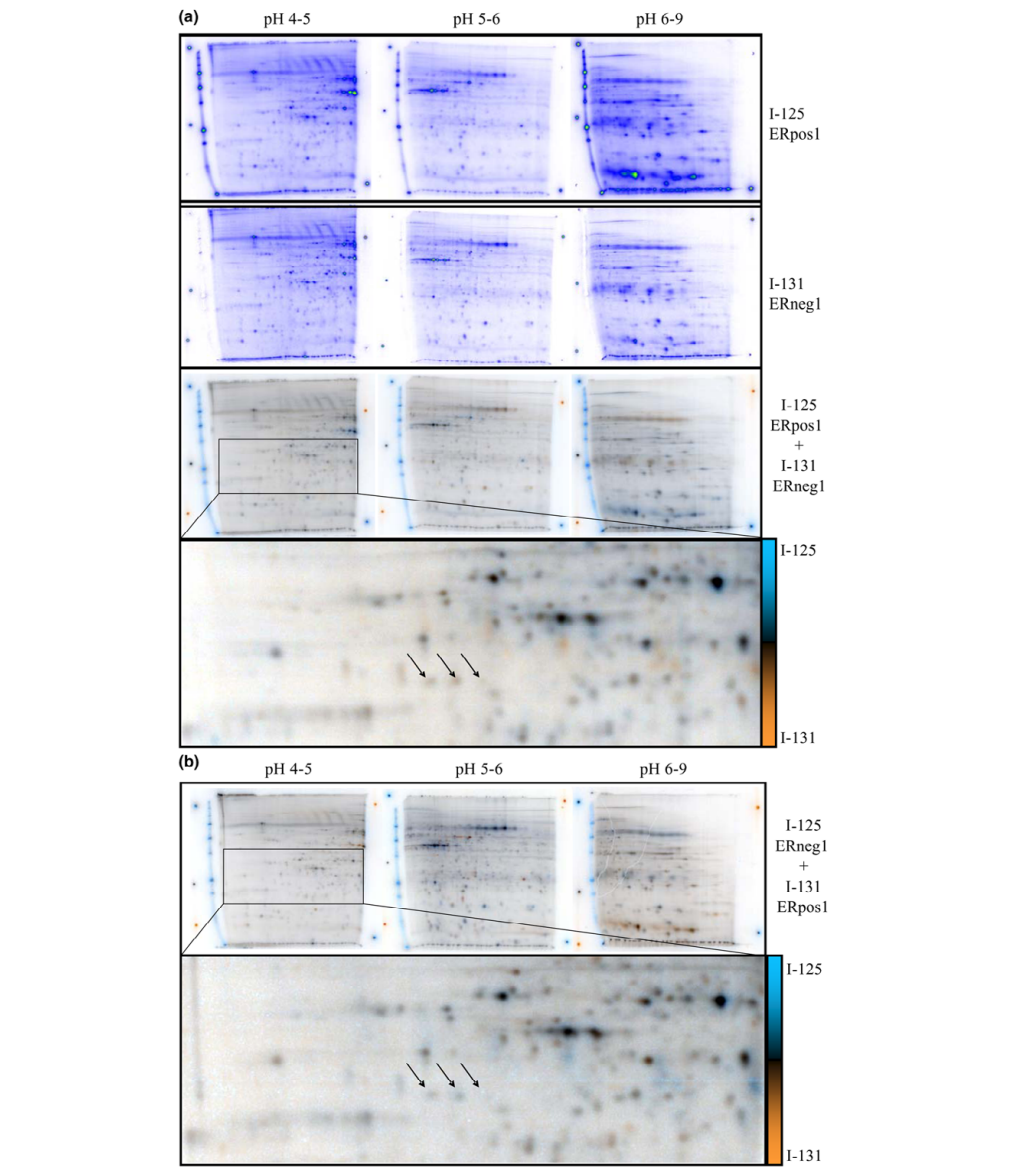
Fifty-four centimeter differential ProteoTope analysis. The panels show actual images from the inverse replicate labeled ProteoTope comparison of all tumors pooled. **(a) **Inverse ProteoTope comparison of all ER-positive samples pooled into one sample (ER+1 to ER+4 from Table 1) co-electrophoresed with pooled all ER-negative samples (ER-1 to ER-4 from Table 1). Proteins from ER-positive tumors are labeled with I-125, differentially compared with proteins from pooled ER-negative labeled tumors with I-131. The upper panels show the individual signal detected for each isotope, depicted in false spectral color. The signals for each isotope were normalized against each other for total relative intensity in the lower dual channel images, where the signal for I-125 is blue, the signal for I-131 is orange, and equal amounts of both signals produces gray or black signal. Two pure sources each of I-131 and I-125, as well as a 50% mixture of both isotopes, are measured on 2 mm sources pipetted next to each dried gel as imaging controls. The pH ranges of the 18 cm IPGs used for serial IEF are indicated above the panels, and the radioactive iodine isotope signals depicted in each panel are indicated on the right. In this experiment all iodination reactions were performed on 60 μg protein. In the examples shown, the I-125 signal is systematically stronger in all gels (compare lower panels for individual isotopes). Arrows in the enlarged lower panel show three spots identified as PGRMC1. **(b) **The top panels show the inverse replicate experiment of panel a, where sample ER+1 is labeled with I-131, and sample ER-1 is labeled with I-125. The panel presentation otherwise follows that given above for panel a. Similar gels were produced for all corresponding differential analyses depicted in Table 1. ER, estrogen receptor; IEF, isoelectric focusing; IPG, immobilized pH gradient; PGMRC, progesterone receptor membrane component.

**Figure 2:**
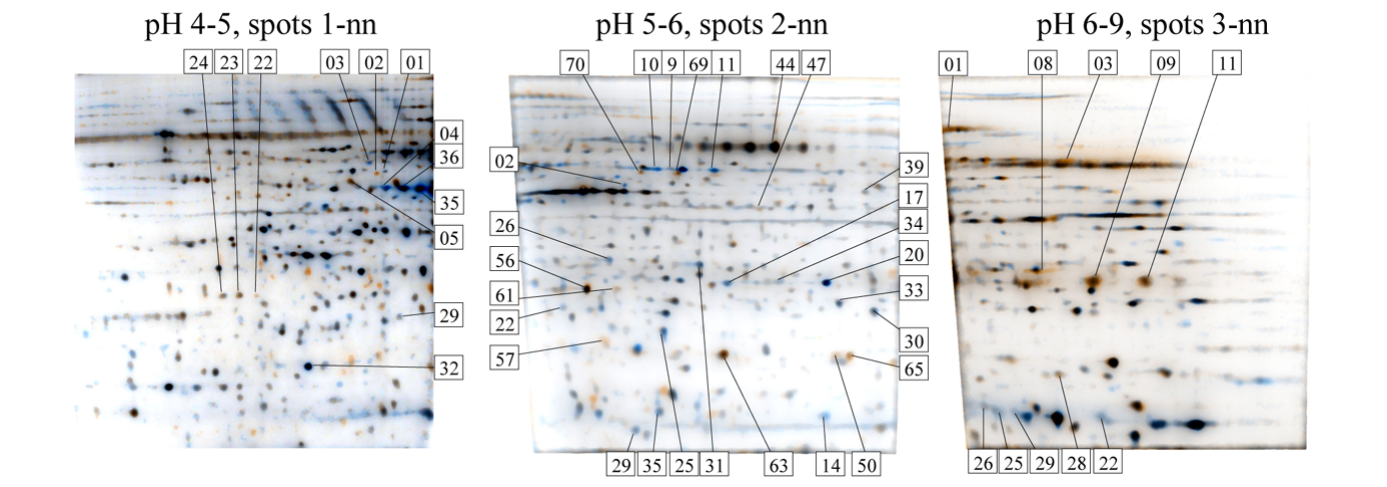
Synthetic average composite gels showing spots matched across all eight IEF gels (24 SDS-PAGE gels). Images were generated using the GREG software and labels were added manually. The average ER-positive signal is indicated as blue, the average ER-negative signal is indicated as orange, and equal intensities of both signals give gray or black pixels. Spot numbers correspond to Figure 3, whereby spots from pH4-5 gel are designated with spot numbers 1-nn, spots from pH5-6 gel are designated with spot numbers 2-nn, and spots from pH6-9 gel are designated with spot numbers 3-nn. For example, PGRMC1 spot 1–24 from Figure 3 is the left-most spot labeled in the pH4-5 gel. Colored signals without spot labels were either unidentifiable or were not consistently differential, with average intensities being markedly influenced by one or few samples and thereby failing to achieve statistical differential significance across the dataset. ER, estrogen receptor; IEF, isoelectric focusing; PGMRC, progesterone receptor membrane component.

**Figure 3:**
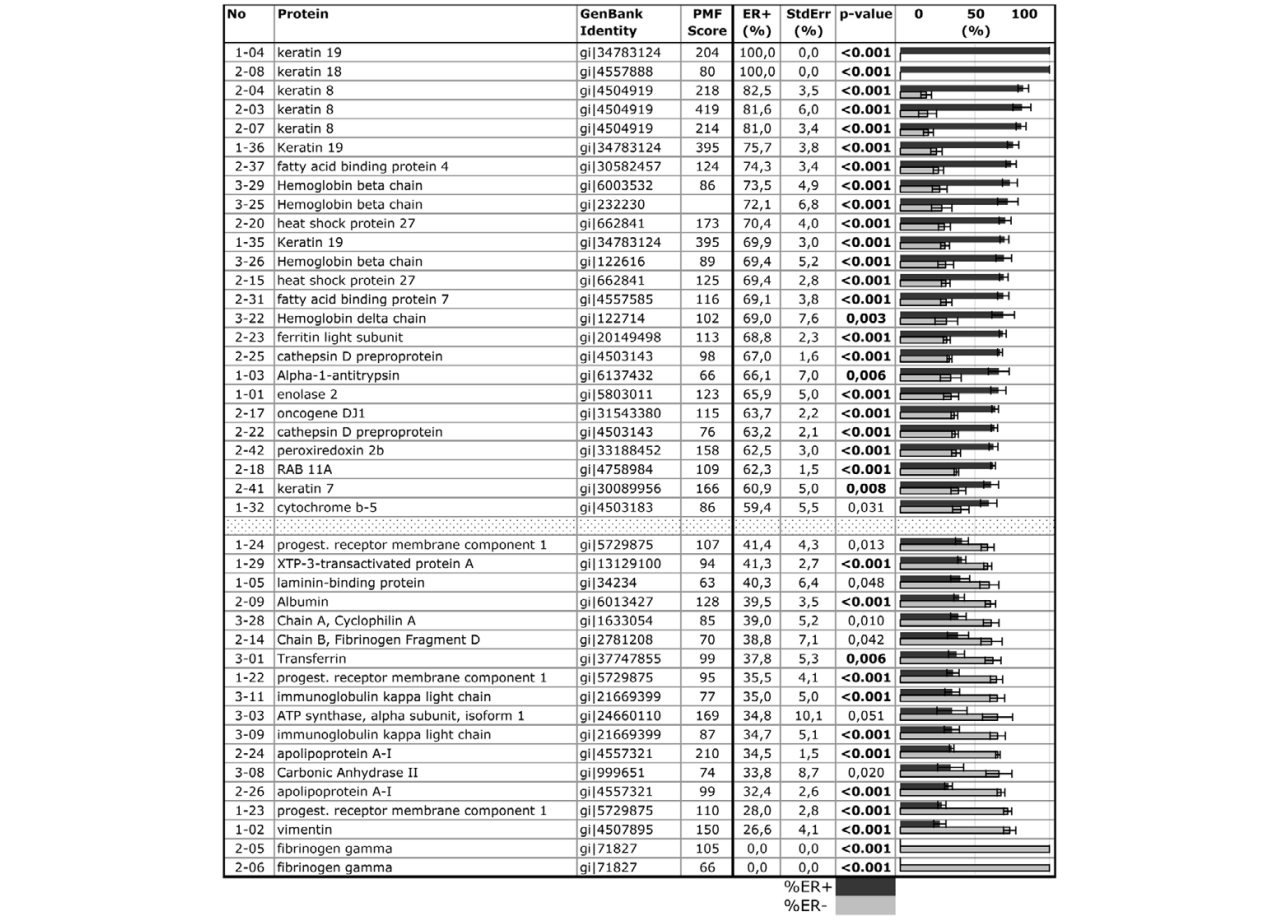
Protein spot quantification and identifications for breast cancer samples (whole tumor slices): ER positive versus ER-negative samples. Spot numbers correspond to those in Figure 2. 'n.i.' means 'not identified'. Genbank Identities are from the NCBI database (version of 4 April 2004). Matrix-assisted laser desorption ionization time of flight (MALDI-TOF) peptide mass fingerprinting (PMF) scores are from MASCOT. The average spot fraction for ER-positive and ER-negative are given as percentage of the normalized total spot volume for each spot (= [ER-positive × 100%]/[ER-positive + ER-negative]) across all patient pools based on two-color ProteoTope analysis for the indicated most significant protein spots. These values were obtained using a least square fit for a model based on all replicates and attributing pool variability as a random effect. The t-test *P *value for this model is also given. *P *values < 0.01 are bold, and *P *values < 0.001 are designated as such. The bars at the right depict average percent abundance of each protein across the ER-positive (black) and ER-negative (gray) pools as indicated above the column with bars (0% to 50% to 100%). Error bars show standard error of means. Protein spots between numbers 1–32 and 1–24 (indicated by a patterned field) are not presented, having failed to meet selection criteria of either abundance difference ratio of 1.5 or significance at the 5% level. ER, estrogen receptor; NCBI, National Center for Biotechnology Information.

We applied a sample pooling strategy to the analysis of clinical protein samples, which permitted generation of effective results from limited amounts of sample [19]. Eight ER-α-positive tumors and eight ER-α-negative tumors were randomly assigned to the subpools summarized in Table 1, each subpool containing normalized equal amounts of protein from two tumors. Pooled samples were differentially quantified according to the regimen summarized in Table 1 (subpool ER+1 [containing T378 and T392] was differentially compared with subpool ER-1 [containing T433 and T443], ER+2 was compared with ER-2, ER+3 was compared with ER-3, and ER+4 was compared with ER-4). Thus, there were four differential comparisons, each performed in inverse replicate 54 cm serial IEF-IPG to generate eight IEF samples and 24× two-dimensional PAGE gels, which were differentially quantified by ProteoTope imaging. Additionally, proteins from all eight tumors from each category were pooled into two master pools, which were also compared by ProteoTope (as presented in Figure 1). Similar gel sets were performed for the paired tumor pools in Table 1 and quantified. Spots were matched across gels, and their intensities were analyzed relative to ER-α status. Synthetic average gel images of the comparisons of pools from Table 1 were constructed by computer, as shown in Figure 2. The two-dimensional PAGE positions of the statistically most significant differential protein spots identified by mass spectrometry are indicated in Figure 2, and their identities are shown in Figure 3. In total, proteins from 325 spots were identified by matrix-assisted laser desorption ionization time of flight (MALDI-TOF) PMF with MASCOT scores greater than 70, of which 72 spots represented 16 proteins that were identified in more than one protein spot (Additional file 1 [Table S1]).

**Table 1:** Pooling design for ER-positive versus ER-negative cryogenic whole tumor sections

Experimental variable designation	Pools	Tumor number	RNA quality	Tumor status	Lymph node status	Grade	ER status	PR status	Her2/neu status	Age of patient (years)
ER positive		T378	ok	2	0	2	12	4	0	75
	ER+1									
		T392	ok	2	0	2	12	2	2^a^	61
	
		T460	ok	2	0	2	4	8	3	79
	ER+2									
		T464	ok	2	0	2	12	8	0	50
	
		T288	ok	2	0	2	12	4	1	76
	ER+3									
		T711	ok	4	2	3	8	6	0	65
	
		T712	ok	2	1	2	9	4	0	58
	ER+4									
		T425	ok	2	1	2	12	0	0	78

ER negative		T433	ok	2	0	2	0	0	1	42
	ER-1									
		T443	ok	2	1	2	0	12	0	46
	
		T469	ok	1	0	2–3	0	0	3	50
	ER-2									
		T470	ok	2	1	2	0	0	0	39
	
		T531	ok	2	0	2	0	0	0	58
	ER-3									
		T558	ok	2	0	2–3	0	0	0	62
	
		T623	ok	1	x	2–3	0	0	1	42
	ER-4									
		T640	ok	2	0	3	0	0	3	62

Individual tumors are designated by their tumor bank T registration numbers. Experimental, clinical, and histopathological parameters are listed. Eight ER-positive and eight ER-negative tumors are grouped into four pools of two tumors each, as indicated. Clinical data comprise the following: tumor status ranging from pT1 (tumor 2 cm or smaller in greatest dimension) to pT3 (tumor >5 cm); lymph node status from pN0 (no regional lymph node metastasis) to pN3 (metastasis to ipsilateral internal mammary lymph nodes [s]) and pNx (regional lymph node cannot be assessed); tumor grade from 2 (moderately differentiated) to 3 (poorly differentiated); histopathologic data for ER and PR (0 = undetectable; 1 to 3 = weakly positive; 4 to 7 = moderately positive; 8–12 = highly positive); and Her2/neu-status (0 = negative, positive +1 to +3). Ages for each patient are given in years. ^a^No gene amplification detected. x, pNx regional lymphnodes cannot be assessed. ER, estrogen receptor; HER, human epidermal growth factor receptor; PR, progesterone receptor.

The differential results observed for this study (Figure 3) provided a protein profile that was consistent with published studies on this clinical system [23-25]. The profile of differentially abundant proteins detected between ER-α-positive and ER-α-negative tumors (Figure 3) shares similarity with the recently reported gene expression profile identified as being specific for the wound response reported for ER-α-negative tumors [17,26]. In the wound response, a genetic program is activated when cells within a tissue are exposed to serum proteins, indicative of permeabilization of the vascular endothelium and local injury. This expression profile provides an index for the extent of wound healing activity in cancers, and this correlates negatively with overall survival and positively with the incidence of metastasis [27,28]. Our results are consistent with the previously reported activated wound response in ER-α-negative tumors. In particular, CK-8, cathepsin B, heat shock protein 27, and ferritin light chain were less abundant in ER-α-negative tumors than in ER-α-positive tumors, whereas vimentin, apolipoprotein A1, cyclophin A, transferrin, carbonic anhydrase, and PGRMC1 were more abundant. This is reminiscent of the wound response signature reported by Velardo and coworkers [26], in which PGRMC1 was upregulated late in the response (presented in Supplementary Table 1 of the report by Velardo and coworkers; AI010357 EST [VEMA] ventral midline antigen = PGRMC1). The serum proteins apolipoprotein A-I and albumin were recently found to be more abundant in a proteomic analysis of injured spinal cord tissue, whereas heat shock protein 27 was found to be downregulated in this wound response relevant system [29]. Taken together, these data indicate that the number of patients analyzed suitably identified differences in protein abundances that were strongly correlated with the presence of the ER-α, indicating that our experimental system yielded results that reflected the biology underlying the ER-α-positive versus ER-α-negative test design.

### PGRMC1 is more abundant in ER-α-negative tumors

PGRMC1, which had not previously been directly associated with ER-α status in breast cancer, was detected in three separate spots. Two of these were significantly more abundant in ER-α-negative tumors (Figure 3), and these were the more basic two spots (Figure 2).

### Two-dimensional PAGE isoforms of PGRMC1 differ in phosphorylation status

**Figure 4:**
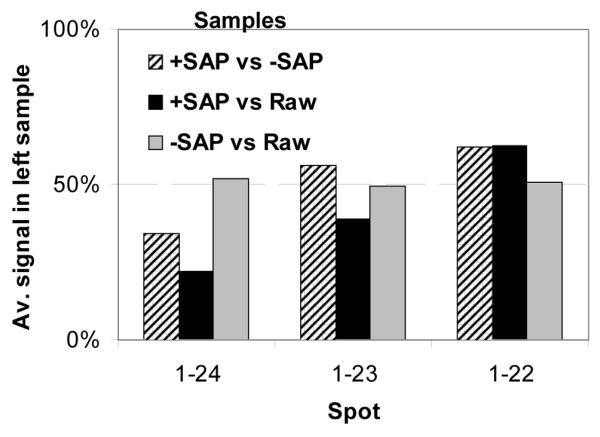
PGRMC1 isoforms in breast tissue differ in phosphorylation status. Shown are differential inverse replicate ProteoTope quantification of PGRMC1 spots 1–22, 1–23, and 1–24 from Figure 2 and Figure 3 from SAP-treated and control samples. The original sample represents proteins pooled from several primary breast tumors for experimental treatment. The treatment nomenclature is as follows: phosphatase treated sample (+SAP), mock incubation control with phosphatase inhibitors and without addition of SAP (-SAP), and original sample frozen before incubation (Raw). Spot numbers are indicated. Quantification of the differential ratio of signal intensities from two samples per two-dimensional PAGE gel for each of the spots from the indicated inverse replicate gel pairs +SAP versus -SAP, +SAP versus Raw, and -SAP versus Raw. The ratio of signal for control and treated samples increases in a phosphatase-dependent manner, consistent with at least some fraction of spots 1–24 and 1–23 in the original sample representing phosphorylated isoforms of PGRMC1 present in spot 1–22. PGMRC, progesterone receptor membrane component; SAP, shrimp alkaline phosphatase.

To assess whether differences in distinct two-dimensional PAGE spot isoforms were due to distinctly phosphorylated species of PGRMC1, we treated proteins from primary breast tumors with SAP and quantified differences in protein isoform abundances using inverse-replicate ProteoTope (Figure 4). Importantly, SAP-dependent differences in relative signal intensity were reproducibly detected in inverse replicate labeled experiments, in which the intensity of spot 1–24 was reduced upon SAP treatment, and the intensity of spot 1–22 increased after SAP treatment. The middle spot 1–23 exhibited variable abundance changes, perhaps because of experimental variation. By contrast, when the mock treatment was compared with the raw extract, the ratios between both samples approximated 1:1. Qualitatively similar overall results were observed in an independent replicate (data not shown). Thus, the differences in intensity of respective spots between samples were not due to the incubation, but rather were due to the presence of phosphatase activity in the incubation mixture. This result demonstrates that the most acidic PGRMC1 spots can be dephosphorylated, whereupon they migrate to one of the more basic spots in two-dimensional PAGE. Taken together with the results presented in Figure 3 for these three protein spots, this provides evidence that PGRMC1 is more highly phosphorylated in ER-α-positive than in ER-α-negative tumors. Because phosphatase treatment did not totally eliminate any PGRMC1 spots, it is possible that protein species within these two-dimensional PAGE spots may also differ by modifications other than phosphorylation.

### Phosphorylation site mutants of PGRMC1 can affect cell survival

**Figure 5:**
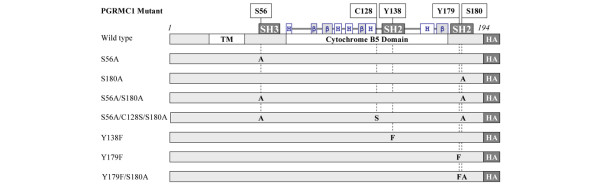
PGRMC1 expression plasmids expressing wild-type PGRMC1 and various amino acid mutants. Mutations were introduced by polymerase chain reaction, after which the entire open reading frame of product plasmids was confirmed correct by DNA sequencing. Individual codon mutations were encoded by the following sequences: serine-56 to alanine (S56A) AGC → GCC; cysteine-128 to serine (C128S) TGC → AGC; tyrosine-138 to phenylalanine (Y138F) TAC → TTC; tyrosine-179 to phenylalanine (Y179F) TAC → TTC; and serine-180 to alanine (S180A) TCA → GCA. Amino acid numbering is according to human PGRMC1 Uniprot O00264, which does not include the initiator methionine. Uniprot Q6IB11 corresponds to the same sequence with initiator methionine included, in which mutated human PGRMC1 amino acids would be numbered as S57, C129, Y139, Y180, and S181. The position of three tandem influenza virus hemaglutinin epitope sequences (HA) is indicated at the carboxyl-terminus of the open reading frame. PGMRC, progesterone receptor membrane component.

Based upon some of the predicted and observed phosphorylation sites for PGRMC1 [20,30,31], we constructed a panel of HA-tagged PGRMC1 expression plasmids based upon pcDNA3_MPR_3HA [32], with amino acid substitutions at the positions of serine-56, serine-180, tyrosine-138, and tyrosine-179 (Figure 5). Because of the proposed role of disulfide bridging to form a 56 kDa dimeric form of PGRMC1, one of the mutants also involved substitution of the conserved cysteine-128 to serine. This residue is the only cysteine in the human PGRMC1 cytochrome b5 domain and is the only phylogenetically conserved cysteine in the protein. On the surface of the protein at the carboxyl-terminal end of helix 3, this cysteine is ideally situated on the protein to physically interact with other proteins (Figure 5) [20].

**Figure 6:**
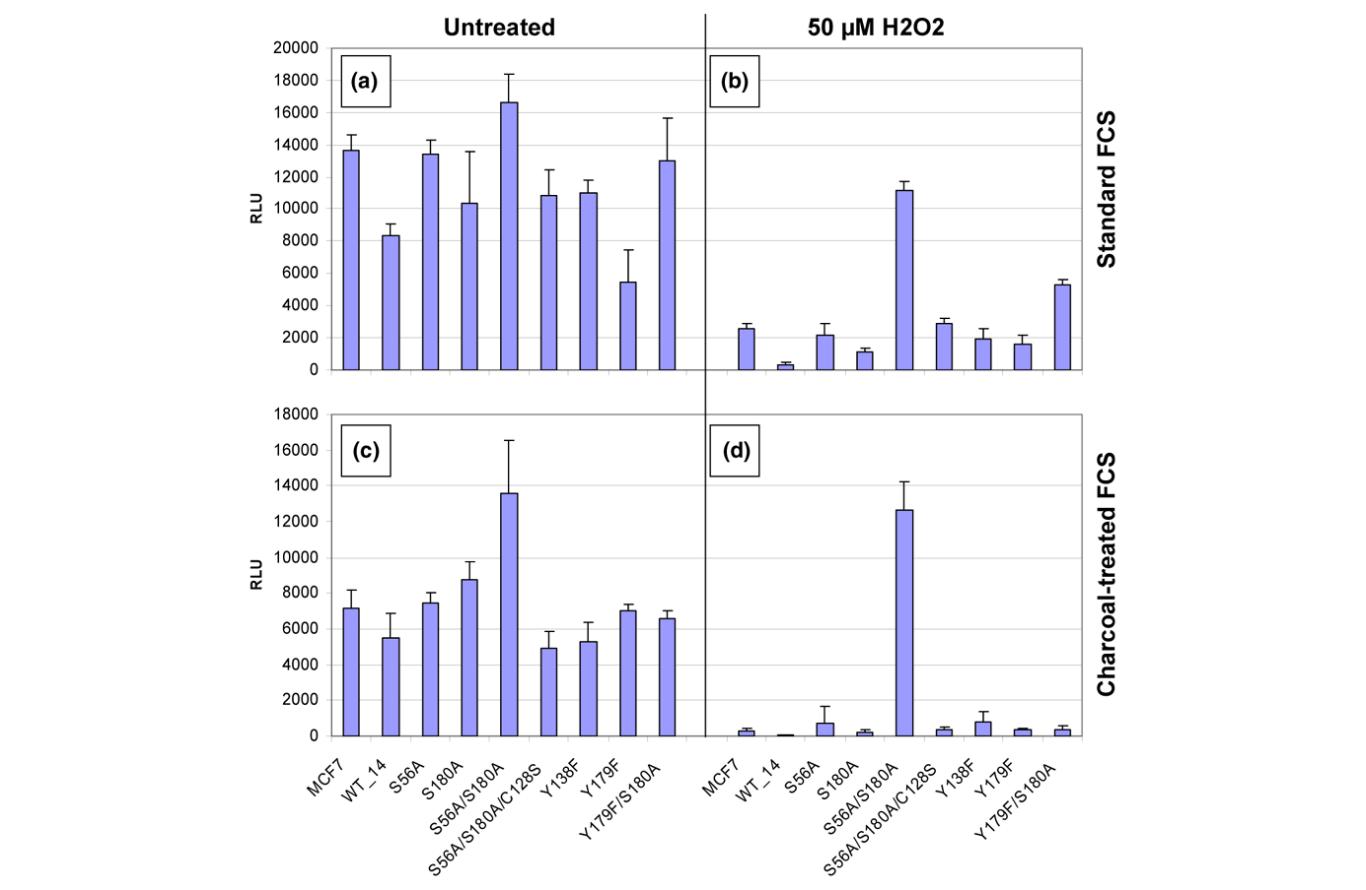
Susceptibility of stably transfected PGRMC1 MCF-7 cell lines to H_2_O_2 _treatment. **(a) **Cell lines are grown in 10% FCS without H_2_O_2 _stress. RLU, relative light units (which reflect the relative ATP content of surviving cells by cell viability assay). **(b) **Cell lines are grown in 10% FCS with the indicated peroxide stress. **(c) **Cell lines are grown in 10% charcoal-treated FCS without H_2_O_2 _stress. **(d) **Cell lines are grown in 10% charcoal-treated FCS with H_2_O_2 _stress. FCS, fetal calf serum; PGMRC, progesterone receptor membrane component.

**Figure 7:**
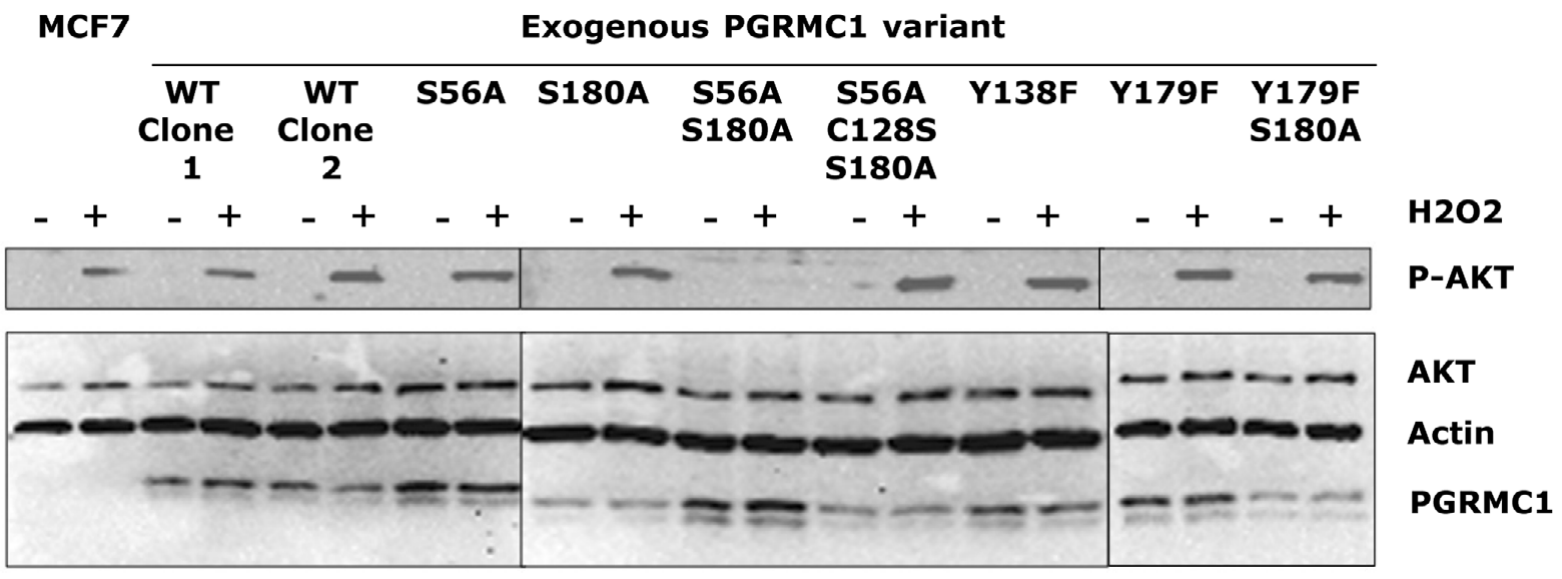
PGRMC1 influences phosphorylation of Akt after H_2_O_2 _treatment. The figure shows a Western blot of equal total protein amounts (20 μg/lane) from cell extracts of the indicated cells. Antibodies employed were specific for phosphorylated Akt (top panel); for the polypeptide backbone of Akt, actin; or for the HA-tag on the respective exogenously expressed PGRMC1 constructs, as indicated. Incubation with the respective antibodies was perfomed simultaneously for the lower panel. Previous experiments indicated no overlap of signal (data not shown). Akt is phosphorylated upon H_2_O_2 _exposure in susceptible MCF-7 cells stably transfected with wild-type PGRMC1 and the mutants indicated. In peroxide-resistant cells expressing the S56A/S180A mutant, Akt phosphorylation is always abrogated. In some experiments in which the Y179F/S180A mutant exhibited limited survival to peroxide (for example, Figure 6b this was also accompanied by lack of Akt phosphorylation (data not shown). These trends were also observed at a concentration of 100 μmol/l H_2_O_2 _with lower overall viability levels (data not shown). PGMRC, progesterone receptor membrane component; HA, hemaglutinin.

Stable expression of PGRMC1 (Hpr6.6) enhances the susceptibility of MCF-7 breast cancer cells to lethality caused by H_2_O_2 _exposure [22]. We transfected MCF-7 cells with each of the PGRMC1 expression plasmids from Figure 5 and established stable transfectant cell lines. Expression of exogenous PGRMC1 was confirmed in all cell lines using an anti-HA tag antibody. The different cell lines exhibited grossly similar cell survival in medium containing 10% FCS (Figure 6a). Upon treatment with 50 μmol/l H_2_O_2_, the viability of untransfected control cells was reduced, and the expression of wild-type PGRMC1 greatly sensitized cells to H_2_O_2 _stress, resulting in lower cell viability (Figure 6b), as expected. Expression of many of the mutant PGRMC1 proteins produced viability comparable with that of nontransfected MCF-7 control cells. However, two of the cell lines (S56A/S180A and Y179F/S180A) exhibited higher viability levels than native MCF-7 cells. The S56A/S180A-expressing cells appeared impervious to the effects of H_2_O_2_, whereas the response of Y179F/S180A-expressing cells varied from marginal survival rates (Figure 6b) to no enhanced survival in other experiments (Figure 7; data not shown).

The above experiment was performed with replicate design but using charcoal/dextran-treated 10% FCS. Charcoal-stripping removes steroid hormones and other hydrophobic components such as cholesterol from the FCS. All cell lines were able to grow in this medium (Figure 6c); however, the degree of viability was greatly impaired in the presence of H_2_O_2 _for all cell lines except the S56A/S180A double mutant (Figure 6d). Therefore, charcoal pretreatment of FCS possibly removes some component that is necessary for the marginal survival of PGRMC1 mutant Y179F/S180A but not for S56A/S180A.

Hand and Craven [22] also observed phosphorylation of the kinase Akt upon H_2_O_2_-induced death of MCF-7 cells; we therefore assayed the degree of Akt phosphorylation in these cells by Western blot. As expected, based on the work reported by Hand and Craven [22], control MCF-7 cells or cells stably expressing exogenous wild-type PGRMC1 exhibited an increase in Akt phosphorylation that correlated with reduced viability after H_2_O_2 _exposure. However, the S56A/S180A mutant that was able to survive peroxide treatment also did not exhibit marked phosphorylation of Akt (Figure 7). In some experiments the Y179F/S180A mutant also exhibited partially reduced levels of Akt phosphorylation (data not shown). Taken together, these data are consistent with differences in the phosphorylation status of PGRMC1 observed in breast cancers, potentially being able to influence the clinically relevant survival phenotype of those cancers. However, further experiments will be necessary to confirm this hypothesis and to elucidate the mechanisms involved.

### PGRMC1 location in tumors

**Figure 8:**
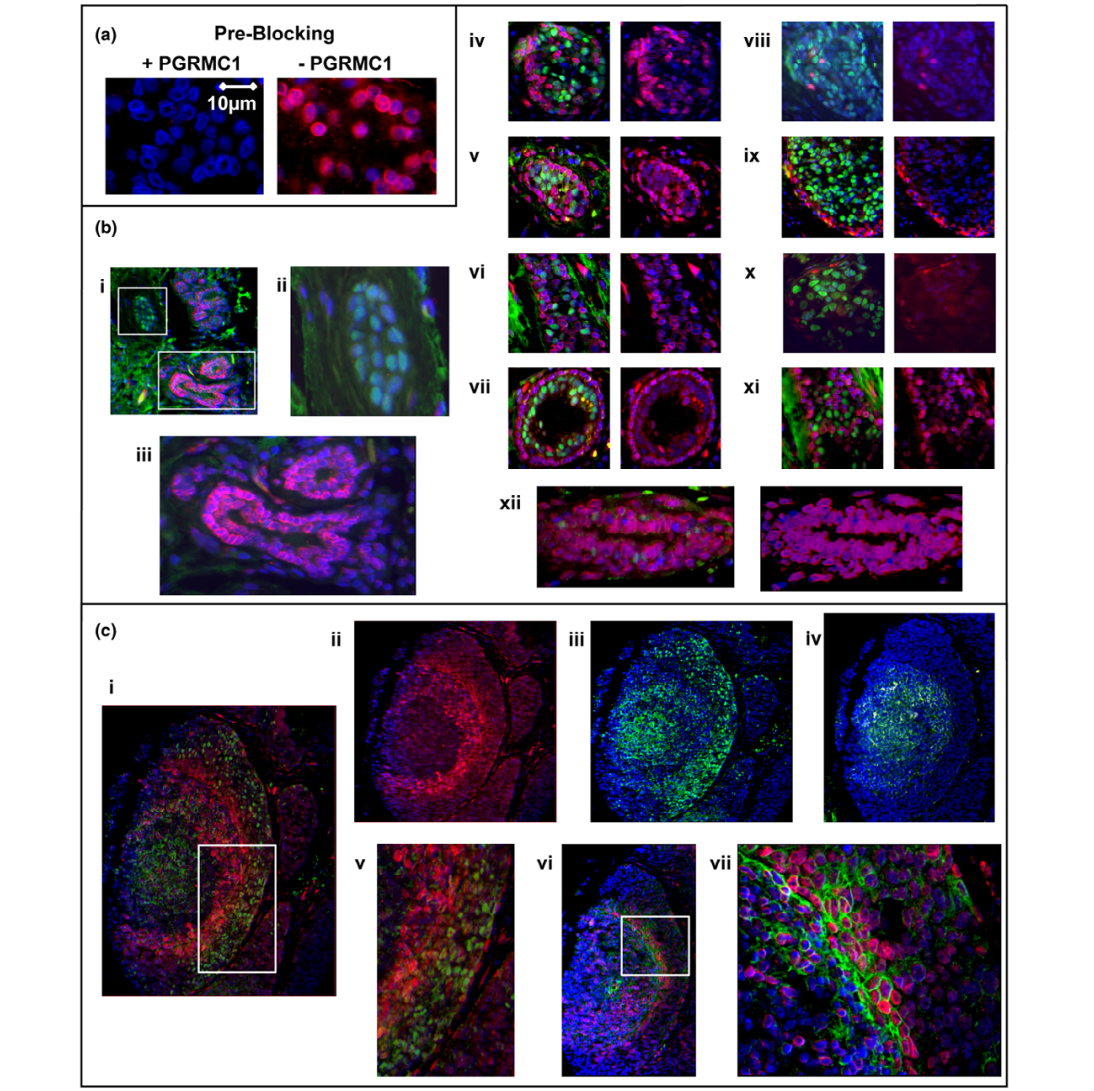
Location of endogenous PGRMC1 in breast cancer tissue. **(a) **Validation of PGRMC1-specific mouse monoclonal antibody 5G7. Breast cancer tissue was labeled with 5G7 with (left) or without (right) prior incubation of the antibody with recombinant PGRMC1 protein. Preincubation of the 5G7 with Δ43hpr6, the cytoplasmic domain of PGRMC1 protein, which served as the immunogen for 5G7, blocks specific detection of PGRMC1 (red). DAPI is used to detect cell nuclei (blue). Magnification: 63×. **(b) **Differential expression of PGRMC1 in ER-α-positive and ER-α-negative tumor cells. Depicted are 10 different breast cancer tissue samples (in subpanels i to xii) from a tissue microarray labelled for ERα (green) and PGRMC1 using monoclonal antibody 5G7 (red). (i) An ER-α-positive invasive ductolobular breast cancer (magnification: 10×). (ii) Higher magnification of upper boxed area in subpanel i, showing an ER-α-positive duct. (iii) Higher magnification of lower boxed area of subpanel i, showing an ER-α-negative duct. Subpanels iv to xii show nine different breast cancer tissues; on the right is shown the red signal without the green signal from the same image. Magnification: 20×. (iv) ER-α-positive invasive ductal carcinoma. (v) ER-α-negative invasive lobular breast cancer. (vi) ER-α-positive invasive ductolobular breast cancer. (vii) ER-α-negative invasive ductal carcinoma. (viii) ER-α-positive invasive ductal carcinoma. (ix) ER-α-positive invasive ductal carcinoma. (x) ER-α-positive ductal carcinoma *in situ*. (xi) ER-α-positive invasive ductal carcinoma. (xii) ER-α-negative invasive ductal breast cancer. The figures indicate that PGRMC1 is differentially expressed in ER-α-positive and ER-α-negative tumor cells. DAPI (blue) is used to detect cell nuclei. **(c) **Differential expression of PGRMC1 in ER-α-positive invasive ductolobular breast cancer sample with a DCIS (comedo type). (i) The tissue is labeled for ER-α (green) and PGRMC1 using ant-PGRMC1 monoclonal antibody 5G7 (red). PGRMC1 is expressed in areas in which ER-α is not expressed. Magnification: 20×. (ii and iii) Depicted is the area of subpanel i with reduced green (ii) or red signal (iii). (iv) Shown is the negative control for subpanels i to iii, using an unlabeled consecutive section, indicating autofluorescence especially in the necrotic center. (v) the boxed area of subpanel i is enlarged. (vi) Depicted is the same ductal carcinoma *in situ *in a consecutive section labeled for glucose transporter (GLUT)-1 (green) and PGRMC1 using 5G7 monoclonal antibody (red). (vii) the boxed area of subpanel vi is enlarged. PGRMC1 is expressed in GLUT-1-positive, hypoxic cells in a perinuclear fashion. GLUT-1 protein is localized at the cytoplasmic membrane. This sample was taken from an ER-α-positive invasive ductolobular breast cancer with *in situ *carcinoma components of comedo-type, and the results depicted were typical for 5/5 comedo type tumors in the stained tissue array. Clinical patient data for the tumors depicted in panels b and c are provided in Additional file 1 (Table S2). DCIS, ductal *in situ *carcinoma; ER, estrogen receptor; PGMRC, progesterone receptor membrane component.

We generated a new specific monoclonal antibody (5G7) against the cytoplasmic domain of PGRMC1 lacking the amino-terminal first 46 amino acids. This antibody recognized endogenous PGRMC1 in breast cancer tissues (Figure 8). The PGRMC1 signal (red in Figure 8) could effectively be abrogated by competitive pre-incubation of the antibody with recombinant PGRMC1 protein (Figure 8a). Co-incubation of anti-ER-α antibody (green signal) and anti-PGRMC1 antibody (red) revealed that these proteins were predominantly expressed in different cells, even in ER-α-positive tumors. Remarkably, very few individual cells were observed that exhibited abundant levels of both ER-α and PGRMC1 (yellow in Figure 8b). In ductal *in situ *breast cancers of comedo-type, PGRMC1 was present in cells surrounding the necrotic centre of the tumor, whereas ER-α was expressed in cells more distal to the necrotic centre (Figure 8c, i to 8c, v). The green fluorescence in the comedo necrotic zone was due to autofluorescence of necrotic cellular material (Figure 8c, iv). The cells expressing PGRMC1 were presumably in the hypoxic zone, and so we performed co-immunofluorescence labeling with 5G7 anti-PGRMC1 and anti-GLUT-1, a hypoxia-inducible factor (HIF)-1 inducible marker for hypoxic cells [33]. Although not all PGRMC1-expressing cells expressed GLUT-1, the vast majority of GLUT-1-positive cells co-expressed PGRMC1. Where PGRMC1 and GLUT-1 proteins were expressed in the same cells, PGRMC1 exhibited a perinuclear location that contrasted markedly with the cytoplasmic membrane localization of GLUT-1 (Figure 8c, vi to 8c, vii). This result provides confirmation of the cellular location of PGRMC1 that was observed for over-expressed HA-tagged PGRMC1/Hpr6 [22].

## Discussion

### Validation of the differential abundance profile

Despite the small number of well characterized tumors employed in this analysis, the sample size is sufficient to detect marked and consistent differences between the test classes with reliable significance. A discussion of the protein abundance profile obtained is provided in Additional file 1. Taken together, these results suggest that our comparison of just eight patients from each group of ER-α-positive or ER-α-negative tumors (Table 1 and Figure 3) has provided useful results that grossly reflect the known differences in biology between these cell types. Therefore, previously unreported protein differences were of extreme interest.

### PGRMC1 and cancer implications

We demonstrate a higher abundance of hypophosphorylated PGRMC1 isoforms in the specific subpopulation of clinically relevant ER-α-negative cancers. Further studies in a larger patient collective will be necessary to correlate specific PGRMC1 isoforms with other tumor markers in addition to ER-α.

We identified three two-dimensional spots corresponding to PGRMC1 (Additional data file 1 [Table S1] and Figure 3), two of which were significantly more abundant in ER-α-negative tumors (spots 1–22 and 1–23 in Figure 3). Phosphatase treatment of primary breast cancer proteins demonstrated that these different isoforms of PGRMC1 differed at least partly in their phosphorylation status (Figure 4).

PGRMC1 was previously reported to be more abundant in a variety of cancers, including breast cancer (although differential ER-α status was not reported), and a perinuclear localization was suggested to implicate it in a role involving cytochrome P450 activation and steroid metabolism [34]. The differential abundance of PGRMC1 protein between breast cancers of different ER-α status is notable because we previously identified the distantly related cytochrome b5-domain feudesin/SPUF protein and cytochrome b5 itself to have been slightly yet significantly differentially abundant between breast tumors that were all positive for the ER-α but which differed in the expression level of the cytoplasmic progesterone receptor [19]. Indeed, cytochrome b5 was also marginally yet significantly more abundant in the ER-α-positive tumors in our present study (1.2-fold [*P *= 0.03]; spot 1–32 in Figure 3). Hughes and colleagues [35] recently reported that PGRMC1 and a fungal homolog are present in evolutionarily conserved protein complexes with respective members of the cytochrome P450 class of enzymes, including the Cyp51A1 protein, which is involved in the production of cholesterol from lanosterol. Furthermore, they demonstrated that reduction in the level of PGRMC1 mRNA and protein produced an elevation in lanosterol levels. A variety of experiments suggest a role of cholesterol in the biology of PGRMC1, as reviewed by Cahill [20]. The rate-limiting enzyme of the mevalonate pathway leading to cholesterol synthesis is hydroxymethylglutarate-coenzyme A reductase, and this enzyme is both regulated by cholesterol levels [36,37] and is diagnostic of a recently identified class of poor prognosis apocrine breast cancers that were both ER-α and progesterone receptor negative [38].

The results presented in Figure 8 indicate that PGRMC1 is abundantly expressed in a population of ER-α-negative and GLUT-1-positive cells in the hypoxic zone surrounding necrotic tumor tissue. GLUT-1 is a membrane glucose transporter that is important in the enhanced rates of anaerobic metabolism of tumors, known as the Warburg effect [33]. Intriguingly, because not all PGRMC1-positive cells expressed GLUT-1 (Figure 8, vi), the population of PGRMC1-expressing cells may have given rise to those expressing GLUT-1, suggesting avenues for future experimentation.

The GLUT-1 and HIF-1 positive cells occupying the hypoxic tumor microenvironment adjacent to necrotic zones are resilient to chemotherapy and frequently give rise to metastases. Although a literature search revealed no directly reported association between the mevalonate pathway and hypoxia, the Wilm's tumor suppressor protein WT1 is thought to suppress growth by downregulating the mevalonate pathway [39], and the hypoxic expression of WT1 is regulated by HIF-1 [40].

Hypoxic conditions have been shown to promote phenotypic de-differentiation in ductal breast carcinoma *in situ*. In mammary ductal *in situ *breast cancer of comedo-type, ductal carcinoma *in situ *(DCIS) cells surrounding the central necrosis exhibited high HIF-1α protein levels, down-regulated ER-α, and increased expression of the epithelial breast stem cell marker CK-19 [41]. These cells lost their polarization and acquired an increased nucleus/cytoplasm ratio, which are hallmarks of poor architectural and cellular differentiation. CK-19 is one marker for a cell population that contains mammary multipotent progenitor cells [42]. Therefore, hypoxia might induce dedifferentiation of epithelial cells, thereby promoting an aggressive phenotype in breast cancer. The hypoxia-induced downregulation of ER-α expression in DCIS has potential clinical relevance and suggests a reason that some ER-α-positive tumors become resistant to anti-estrogen treatment. Because PGRMC1 is upregulated in the cells close to the necrotic area, it conceivably plays a role in this phenomenon.

HIF-1 also induces the angiogenic growth factor vascular endothelial growth factor [33]. Swiatek-De Lange and colleagues [43] implicated PGRMC1 in the activation of vascular endothelial growth factor gene expression in retinal glial cells. Interestingly, PGRMC1 (AI010357 EST [VEMA] ventral midline antigen) was observed to be one of a number of genes upregulated in the late phase of a wound healing model involving injured spinal cord [26], at a time when vascular morphogenesis occurs in the healing tissue.

PGRMC1 protein affects the response to oxidative damage in the MCF-7 breast cancer cell line, influencing their susceptibility to oxidative cell death [22]. It is unclear whether this reflects a normal function of PGRMC1 or is a function of the conditions of over-expression. However, under these conditions, some of our phosphorylation site PGRMC1 mutants exhibited enhanced survival (Figure 6). Both survival and failure to induce Akt phosphorylation were associated with somewhat higher levels of the exogenous S56A/S180A mutant PGRMC1 protein detected by Western blot (Figure 7), but our data do not demonstrate that this higher level is reproducible, and similar levels of the other mutants did not protect against cell death, suggesting that elevated exogenous PGRMC1 protein abundance levels *per se *were not responsible for enhanced survival of MCF-7 cells expressing the S56A/S180A mutant. Indeed, over-expression of PGRMC1 above endogenous levels increased susceptibility to peroxide-induced death [22] (Figure 7). It is possible that the failure of the S56A/S180A mutant to be phosphorylated on those residues leads to accumulation of some biologically active species that is/are perhaps inappropriately cleared. For instance, sterol levels regulate the ubiquitination and degradation of both Insig-1 and hydroxymethylglutarate-coenzyme A reductase to downregulate the mevalonate pathway [44,45], and PGRMC1 interacts directly with Insig-1 [32].

The possible mechanism of survival of the S56A/S180 mutant deserves some consideration. Phosphorylation of S56 presumably blocks the interaction of PGRMC1 with another protein(s) through the predicted proline-rich SH3 target domain centered on P62, whereas phosphorylation of S181 presumably blocks phosphorylation of the adjacent Y179, which would be necessary for interaction with one or more presumed SH2-domain proteins [20]. Phosphorylation of Y179 probably requires the prior regulatory dephosphorylation of S180. C128 was essential for the vital function of the S56A/S180 mutant, and it is quite possible that dimerization via a cystine-mediated disulfide bond [20] is required for the rescuing function. Mutation of cysteine to serine is unlikely to have greatly affected protein structure. Furthermore, the inability of phosphorylated Y179 to interact with one or more unidentified SH2 domain-containing proteins may be responsible for the susceptibility of the Y179F/S180A to growth in charcoal-treated FCS.

### Candidate PGRMC1-interacting proteins

It is reasonable to speculate that differences in the phosphorylation status of PGRMC1 can affect the proteins with which it interacts, and thereby affect cellular biology. The possible breast cancer relevance of known or suspected interactions of PGRMC1 with PAIRBP1/CGI-55, neogenin and DCC (deleted in colon cancer) are considered in the supplementary discussion included in Additional file 1. Future research should address what role, if any, these proposed interactions of PGRMC1 with those candidate interaction partners may play in breast cancer.

## Conclusions

**Figure 9:**
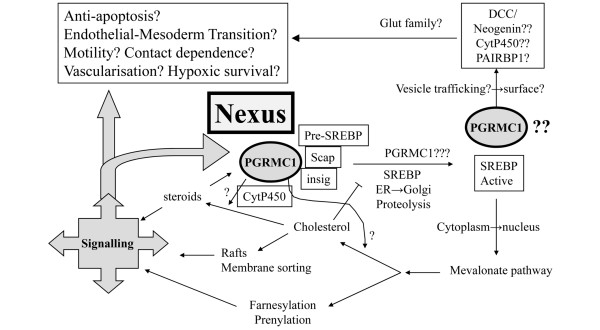
Hypothetical functions of PGRMC1 in cancer biology. PGRMC1 potentially occupies a regulatory nexus; presented is a schematic model for some hypothetical functions of PGRMC1 in cancer biology, many of which require future experimental validation. See also the supplementary discussion in Additional data file 1.

Taken together, this emerging picture strongly suggests that PGRMC1 is potentially able to impinge upon the regulation of cell biology that is centrally important for the clinical consequences of tumors, potentially maintaining not only cell migration and tissue morphogenesis but also tissue homeostasis. There are therefore a variety of theoretically possible mechanisms whereby differential PGRMC1 abundance and phosphorylation could affect tumor biology, perhaps with a central nexus functionality (Figure 9). This work suggests directions for further experiments that will be necessary to address the explicit role(s) of PGRMC1 in cancer.

We detected an expected wound response signature in ER-α-neg tumors that was associated for the first time with differential abundance, and phosphorylation of PGRMC1 between different tumor types. Furthermore, our data suggest that the phosphorylation status of PGRMC1 can affect cell survival in response to life-threatening conditions. Determination of the thus far poorly defined role of PGRMC1 in cancer biology could prove to be of great relevance to clinical cancer therapists. Indeed, relevance to a broad range of tissues and pathologies is quite probable.

## Abbreviations

CK: cytokeratin; DCIS: ductal carcinoma *in situ*; ER: estrogen receptor; FCS: fetal calf serum; GLUT: glucose transporter; HA: hemaglutinin; HIF: hypoxia-inducible factor; IEF: isoelectric focusing; IPG: immobilized pH gradient; PGMRC: progesterone receptor membrane component; PMF: peptide mass fingerprinting; RPMI: Roswell Park Memorial Institute; SAP: shrimp alkaline phosphatase.

## Competing interests

In line with the policies of the German Human Genome Project (DHGP), patent applications (WO2006029836, WO2007039189, and EP1938103) have been filed regarding these results, of which SC, HN, DW, RK, and MAC are inventors. If commercial profits are generated therefrom, then ProteoSys AG, the University of Tübingen, and SC will be financial beneficiaries. WW, GPS, SP, WS, and MAC were employees of ProteoSys AG during all or some of the period of this study. WS and MAC are stockholders of ProteoSys AG.

## Authors' contributions

HN, SEC, and RK were involved in conception and design, acquisition of data (clinical), and analysis and interpretation of data. WW, SP, and WS were involved in the acquisition of data (proteomics). GSP was involved in involved in conception, design, and acquisition of data (proteomics). KS and UV were involved in acquisition of data (histology). TF and DW were involved in conception and design (clinical tumor samples). MAC was involved in conception and design (proteomics and some clinical), analysis and interpretation of data, and was also the main author of the manuscript.

## Supplementary Material

Additional file 1 Supplementary materials. Presented are Supplementary discussions entitled 'Validation of the differential abundance profile' and 'Candidate PGRMC1 interacting proteins'. It additionally contains two supplementary tables: Table S1 ('Protein spots that contained multiple identifications of individual proteins as gene products') and Table S2 ('Clinical patient data for the tumours in Figure 8B and 8C'). Click here for file

## References

[B1] Jemal A, Siegel R, Ward E, Hao Y, Xu J, Murray T, Thun MJ (2008). Cancer statistics, 2008. CA Cancer J Clin.

[B2] Fisher B, Jeong JH, Dignam J, Anderson S, Mamounas E, Wickerham DL, Wolmark N (2001). Findings from recent National Surgical Adjuvant Breast and Bowel Project adjuvant studies in stage I breast cancer. J Natl Cancer Inst Monogr.

[B3] Osborne CK (1998). Steroid hormone receptors in breast cancer management. Breast Cancer Res Treat.

[B4] Henderson IC (1993). Risk factors for breast cancer development. Cancer.

[B5] Howell A, Osborne CK, Morris C, Wakeling AE (2000). ICI 182,780 (Faslodex): development of a novel, 'pure' antiestrogen. Cancer.

[B6] McKeage K, Curran MP, Plosker GL (2004). Fulvestrant: a review of its use in hormone receptor-positive metastatic breast cancer in postmenopausal women with disease progression following antiestrogen therapy. Drugs.

[B7] Platet N, Prevostel C, Derocq D, Joubert D, Rochefort H, Garcia M (1998). Breast cancer cell invasiveness: correlation with protein kinase C activity and differential regulation by phorbol ester in estrogen receptor-positive and -negative cells. Int J Cancer.

[B8] Thompson EW, Paik S, Brunner N, Sommers CL, Zugmaier G, Clarke R, Shima TB, Torri J, Donahue S, Lippman ME, Martin GR, Dickson RB (1992). Association of increased basement membrane invasiveness with absence of estrogen receptor and expression of vimentin in human breast cancer cell lines. J Cell Physiol.

[B9] Fuqua SA, Cui Y (2004). Estrogen and progesterone receptor isoforms: clinical significance in breast cancer. Breast Cancer Res Treat.

[B10] Price JE, Polyzos A, Zhang RD, Daniels LM (1990). Tumorigenicity and metastasis of human breast carcinoma cell lines in nude mice. Cancer Res.

[B11] Sørlie T, Perou CM, Tibshirani R, Aas T, Geisler S, Johnsen H, Hastie T, Eisen MB, Rijn M van de, Jeffrey SS, Thorsen T, Quist H, Matese JC, Brown PO, Botstein D, Eystein Lønning P, Børresen-Dale AL (2001). Gene expression patterns of breast carcinomas distinguish tumor subclasses with clinical implications. Proc Natl Acad Sci USA.

[B12] Sorlie T, Tibshirani R, Parker J, Hastie T, Marron JS, Nobel A, Deng S, Johnsen H, Pesich R, Geisler S, Demeter J, Perou CM, Lønning PE, Brown PO, Børresen-Dale AL, Botstein D (2003). Repeated observation of breast tumor subtypes in independent gene expression data sets. Proc Natl Acad Sci USA.

[B13] Abd El-Rehim DM, Pinder SE, Paish CE, Bell J, Blamey RW, Robertson JF, Nicholson RI, Ellis IO (2004). Expression of luminal and basal cytokeratins in human breast carcinoma. J Pathol.

[B14] Nielsen TO, Hsu FD, Jensen K, Cheang M, Karaca G, Hu Z, Hernandez-Boussard T, Livasy C, Cowan D, Dressler L, Akslen LA, Ragaz J, Gown AM, Gilks CB, Rijn M van de, Perou CM (2004). Immunohistochemical and clinical characterization of the basal-like subtype of invasive breast carcinoma. Clin Cancer Res.

[B15] Weigelt B, Glas AM, Wessels LF, Witteveen AT, Peterse JL, van't Veer LJ (2003). Gene expression profiles of primary breast tumors maintained in distant metastases. Proc Natl Acad Sci USA.

[B16] Dvorak HF (1986). Tumors: wounds that do not heal. Similarities between tumor stroma generation and wound healing. N Engl J Med.

[B17] Chang HY, Sneddon JB, Alizadeh AA, Sood R, West RB, Montgomery K, Chi JT, Rijn M van de, Botstein D, Brown PO (2004). Gene expression signature of fibroblast serum response predicts human cancer progression: similarities between tumors and wounds. PLoS Biol.

[B18] Poznanovic S, Wozny W, Schwall GP, Sastri C, Hunzinger C, Stegmann W, Schrattenholz A, Buchner A, Gangnus R, Burgemeister R, Cahill MA (2005). Differential radioactive proteomic analysis of microdissected renal cell carcinoma tissue by 54 cm isoelectric focusing in serial immobilized pH gradient gels. J Proteome Res.

[B19] Neubauer H, Clare SE, Kurek R, Fehm T, Wallwiener D, Sotlar K, Nordheim A, Wozny W, Schwall GP, Poznanoviæ S, Sastri C, Hunzinger C, Stegmann W, Schrattenholz A, Cahill MA (2006). Breast cancer proteomics by laser capture microdissection, sample pooling, 54-cm IPG IEF, and differential iodine radioisotope detection. Electrophoresis.

[B20] Cahill MA (2007). Progesterone receptor membrane component 1: an integrative review. J Steroid Biochem Mol Biol.

[B21] Perkins DN, Pappin DJ, Creasy DM, Cottrell JS (1999). Probability-based protein identification by searching sequence databases using mass spectrometry data. Electrophoresis.

[B22] Hand RA, Craven RJ (2003). Hpr6.6 protein mediates cell death from oxidative damage in MCF-7 human breast cancer cells. J Cell Biochem.

[B23] Perou CM, Sørlie T, Eisen MB, Rijn M van de, Jeffrey SS, Rees CA, Pollack JR, Ross DT, Johnsen H, Akslen LA, Fluge O, Pergamenschikov A, Williams C, Zhu SX, Lønning PE, Børresen-Dale AL, Brown PO, Botstein D (2000). Molecular portraits of human breast tumours. Nature.

[B24] van 't Veer LJ, Dai H, Vijver MJ van de, He YD, Hart AA, Mao M, Peterse HL, Kooy K van der, Marton MJ, Witteveen AT, Schreiber GJ, Kerkhoven RM, Roberts C, Linsley PS, Bernards R, Friend SH (2002). Gene expression profiling predicts clinical outcome of breast cancer. Nature.

[B25] Gruvberger S, Ringner M, Chen Y, Panavally S, Saal LH, Borg A, Ferno M, Peterson C, Meltzer PS (2001). Estrogen receptor status in breast cancer is associated with remarkably distinct gene expression patterns. Cancer Res.

[B26] Velardo MJ, Burger C, Williams PR, Baker HV, Lopez MC, Mareci TH, White TE, Muzyczka N, Reier PJ (2004). Patterns of gene expression reveal a temporally orchestrated wound healing response in the injured spinal cord. J Neurosci.

[B27] Bertucci F, Finetti P, Rougemont J, Charafe-Jauffret E, Cervera N, Tarpin C, Nguyen C, Xerri L, Houlgatte R, Jacquemier J, Viens P, Birnbaum D (2005). Gene expression profiling identifies molecular subtypes of inflammatory breast cancer. Cancer Res.

[B28] Chang HY, Nuyten DS, Sneddon JB, Hastie T, Tibshirani R, Sørlie T, Dai H, He YD, van't Veer LJ, Bartelink H, Rijn M van de, Brown PO, Vijver MJ van de (2005). Robustness, scalability, and integration of a wound-response gene expression signature in predicting breast cancer survival. Proc Natl Acad Sci USA.

[B29] Kang SK, So HH, Moon YS, Kim CH (2006). Proteomic analysis of injured spinal cord tissue proteins using 2-DE and MALDI-TOF MS. Proteomics.

[B30] Beausoleil SA, Jedrychowski M, Schwartz D, Elias JE, Villen J, Li J, Cohn MA, Cantley LC, Gygi SP (2004). Large-scale characterization of HeLa cell nuclear phosphoproteins. Proc Natl Acad Sci USA.

[B31] Sauer G, Korner R, Hanisch A, Ries A, Nigg EA, Sillje HH (2005). Proteome analysis of the human mitotic spindle. Mol Cell Proteomics.

[B32] Suchanek M, Radzikowska A, Thiele C (2005). Photo-leucine and photo-methionine allow identification of protein-protein interactions in living cells. Nat Methods.

[B33] Airley RE, Mobasheri A (2007). Hypoxic regulation of glucose transport, anaerobic metabolism and angiogenesis in cancer: novel pathways and targets for anticancer therapeutics. Chemotherapy.

[B34] Crudden G, Loesel R, Craven RJ (2005). Overexpression of the cytochrome p450 activator hpr6 (heme-1 domain protein/human progesterone receptor) in tumors. Tumour Biol.

[B35] Hughes AL, Powell DW, Bard M, Eckstein J, Barbuch R, Link AJ, Espenshade PJ (2007). Dap1/PGRMC1 binds and regulates cytochrome P450 enzymes. Cell Metab.

[B36] Sever N, Song BL, Yabe D, Goldstein JL, Brown MS, DeBose-Boyd RA (2003). Insig-dependent ubiquitination and degradation of mammalian 3-hydroxy-3-methylglutaryl-CoA reductase stimulated by sterols and geranylgeraniol. J Biol Chem.

[B37] Brown AJ, Sun L, Feramisco JD, Brown MS, Goldstein JL (2002). Cholesterol addition to ER membranes alters conformation of SCAP, the SREBP escort protein that regulates cholesterol metabolism. Mol Cell.

[B38] Celis JE, Gromov P, Moreira JM, Cabezon T, Friis E, Vejborg IM, Proess G, Rank F, Gromova I (2006). Apocrine cysts of the breast: biomarkers, origin, enlargement, and relation with cancer phenotype. Mol Cell Proteomics.

[B39] Rae FK, Martinez G, Gillinder KR, Smith A, Shooter G, Forrest AR, Grimmond SM, Little MH (2004). Analysis of complementary expression profiles following WT1 induction versus repression reveals the cholesterol/fatty acid synthetic pathways as a possible major target of WT1. Oncogene.

[B40] Wagner KD, Wagner N, Wellmann S, Schley G, Bondke A, Theres H, Scholz H (2003). Oxygen-regulated expression of the Wilms' tumor suppressor Wt1 involves hypoxia-inducible factor-1 (HIF-1). Faseb J.

[B41] Helczynska K, Kronblad A, Jogi A, Nilsson E, Beckman S, Landberg G, Pahlman S (2003). Hypoxia promotes a dedifferentiated phenotype in ductal breast carcinoma in situ. Cancer Res.

[B42] Gudjonsson T, Villadsen R, Nielsen HL, Ronnov-Jessen L, Bissell MJ, Petersen OW (2002). Isolation, immortalization, and characterization of a human breast epithelial cell line with stem cell properties. Genes Dev.

[B43] Swiatek-De Lange M, Stampfl A, Hauck SM, Zischka H, Gloeckner CJ, Deeg CA, Ueffing M (2007). Membrane-initiated effects of progesterone on calcium dependent signaling and activation of VEGF gene expression in retinal glial cells. Glia.

[B44] Lee JN, Song B, DeBose-Boyd RA, Ye J (2006). Sterol-regulated degradation of Insig-1 mediated by the membrane-bound ubiquitin ligase gp78. J Biol Chem.

[B45] Song BL, DeBose-Boyd RA (2004). Ubiquitination of 3-hydroxy-3-methylglutaryl-CoA reductase in permeabilized cells mediated by cytosolic E1 and a putative membrane-bound ubiquitin ligase. J Biol Chem.

[B46] Page MJ, Amess B, Townsend RR, Parekh R, Herath A, Brusten L, Zvelebil MJ, Stein RC, Waterfield MD, Davies SC, O'Hare MJ (1999). Proteomic definition of normal human luminal and myoepithelial breast cells purified from reduction mammoplasties. Proc Natl Acad Sci USA.

[B47] Narod SA, Foulkes WD (2004). BRCA1 and BRCA2: 1994 and beyond. Nat Rev Cancer.

[B48] Chu PG, Weiss LM (2002). Keratin expression in human tissues and neoplasms. Histopathology.

[B49] Boecker W, Buerger H (2003). Evidence of progenitor cells of glandular and myoepithelial cell lineages in the human adult female breast epithelium: a new progenitor (adult stem) cell concept. Cell Prolif.

[B50] Fan C, Oh DS, Wessels L, Weigelt B, Nuyten DS, Nobel AB, van't Veer LJ, Perou CM (2006). Concordance among gene-expression-based predictors for breast cancer. N Engl J Med.

[B51] Folkman J (1995). Angiogenesis in cancer, vascular, rheumatoid and other disease. Nat Med.

[B52] Garvin S, Nilsson UW, Dabrosin C (2005). Effects of oestradiol and tamoxifen on VEGF, soluble VEGFR-1, and VEGFR-2 in breast cancer and endothelial cells. Br J Cancer.

[B53] Ardelt AA, McCullough LD, Korach KS, Wang MM, Munzenmaier DH, Hurn PD (2005). Estradiol regulates angiopoietin-1 mRNA expression through estrogen receptor-alpha in a rodent experimental stroke model. Stroke.

[B54] Gerner C, Steinkellner W, Holzmann K, Gsur A, Grimm R, Ensinger C, Obrist P, Sauermann G (2001). Elevated plasma levels of crosslinked fibrinogen gamma-chain dimer indicate cancer-related fibrin deposition and fibrinolysis. Thromb Haemost.

[B55] Dvorak HF, Harvey VS, Estrella P, Brown LF, McDonagh J, Dvorak AM (1987). Fibrin containing gels induce angiogenesis. Implications for tumor stroma generation and wound healing. Lab Invest.

[B56] Palumbo JS, Kombrinck KW, Drew AF, Grimes TS, Kiser JH, Degen JL, Bugge TH (2000). Fibrinogen is an important determinant of the metastatic potential of circulating tumor cells. Blood.

[B57] Moroz OV, Murzin AG, Makarova KS, Koonin EV, Wilson KS, Galperin MY (2005). Dimeric dUTPases, HisE, and MazG belong to a new superfamily of all-alpha NTP pyrophosphohydrolases with potential 'house-cleaning' functions. J Mol Biol.

[B58] Peluso JJ, Pappalardo A, Losel R, Wehling M (2005). Expression and function of PAIRBP1 within gonadotropin-primed immature rat ovaries: PAIRBP1 regulation of granulosa and luteal cell viability. Biol Reprod.

[B59] Engmann L, Losel R, Wehling M, Peluso JJ (2006). Progesterone regulation of human granulosa/luteal cell viability by an RU486-independent mechanism. J Clin Endocrinol Metab.

[B60] Lemos TA, Passos DO, Nery FC, Kobarg J (2003). Characterization of a new family of proteins that interact with the C-terminal region of the chromatin-remodeling factor CHD-3. FEBS Lett.

[B61] Zhang H, Stephens LC, Kumar R (2006). Metastasis tumor antigen family proteins during breast cancer progression and metastasis in a reliable mouse model for human breast cancer. Clin Cancer Res.

[B62] Leader JE, Wang C, Popov VM, Fu M, Pestell RG (2006). Epigenetics and the estrogen receptor. Ann N Y Acad Sci.

[B63] Nguyen A, Cai H (2006). Netrin-1 induces angiogenesis via a DCC-dependent ERK1/2-eNOS feed-forward mechanism. Proc Natl Acad Sci USA.

[B64] Runko E, Kaprielian Z (2004). *Caenorhabditis elegans *VEM-1, a novel membrane protein, regulates the guidance of ventral nerve cord-associated axons. J Neurosci.

[B65] Mehlen P, Furne C (2005). Netrin-1: when a neuronal guidance cue turns out to be a regulator of tumorigenesis. Cell Mol Life Sci.

[B66] Bouchard JF, Moore SW, Tritsch NX, Roux PP, Shekarabi M, Barker PA, Kennedy TE (2004). Protein kinase A activation promotes plasma membrane insertion of DCC from an intracellular pool: a novel mechanism regulating commissural axon extension. J Neurosci.

[B67] Krauss RS, Cole F, Gaio U, Takaesu G, Zhang W, Kang JS (2005). Close encounters: regulation of vertebrate skeletal myogenesis by cell-cell contact. J Cell Sci.

[B68] Nikolopoulos SN, Giancotti FG (2005). Netrin-integrin signaling in epithelial morphogenesis, axon guidance and vascular patterning. Cell Cycle.

[B69] Slorach EM, Werb Z (2003). Epithelial morphogenesis: Netrin comes to a sticky and terminal end. Curr Biol.

[B70] Strizzi L, Bianco C, Raafat A, Abdallah W, Chang C, Raafat D, Hirota M, Hamada S, Sun Y, Normanno N, Callahan R, Hinck L, Salomon D (2005). Netrin-1 regulates invasion and migration of mouse mammary epithelial cells overexpressing Cripto-1 in vitro and in vivo. J Cell Sci.

[B71] Huber MA, Kraut N, Beug H (2005). Molecular requirements for epithelial-mesenchymal transition during tumor progression. Curr Opin Cell Biol.

