# Supplementary material for: Breast cancer proteomics reveals correlation between estrogen receptor status and differential phosphorylation of PGRMC1
Source: Breast Cancer Res. 2008 Oct 15;10(5):R85. doi: 10.1186/bcr2155 (PMC2614521; doi:10.1186/bcr2155)
Supplement: Additional file 1 — Supplementary materials. Presented are Supplementary discussions entitled 'Validation of the differential abundance profile' and 'Candidate PGRMC1 interacting proteins'. It additionally contains two supplementary tables: Table S1 ('Protein spots that contained multiple identifications of individual proteins as gene products') and Table S2 ('Clinical patient data for the tumours in Figure 8B and 8C'). [file bcr2155-S1.pdf]

Breast cancer proteomics reveals correlation between Estrogen Receptor status and differential phosphorylation of PGRMC1

Hans Neubauer, Susan E Clare, Wojciech Wozny, Gerhard P Schwall, Slobodan Poznanovic, Werner Stegmann, Ulrich Vogel, Karl Sotlar, Diethelm Wallwiener, Raffael Kurek, Tanja Fehm and Michael A Cahill

Additional data file 1

Supplementary Discussion

Validation of the differential abundance profile

We observed markedly elevated levels of keratins 19, 18 and 8 in ERα-pos tumors, relative to ERα-neg tumors. Most ERα-pos cancers exhibit a phenotype similar to luminal epithelial cells of the milk ducts. Keratins 8 and 18 are characteristic of most secretory cells, and keratins 9 and 19 are present in ductal epithelial cells [46-48], while Cytokeratin 5 and Smooth Muscle Actin are characteristic of ERα-neg myoepithelial basal cells [49]. Our results show that particularly Cytokeratin 8, Cathepsin B, HSP27, and Ferritin Light Chain were less abundant in ERα-neg tumors than ERα-pos tumors, while Vimentin, Cyclophin A, Transferrin, Carbonic Anhydrase, and PGRMC1 as well as Apolipoprotein A1 and Albumin were more abundant in ERα-neg tumors, in accord with the reported wound response signature [26-29].

Fan et al. identified a high concordance among gene expression-based predictors for breast cancer. Therein, four significant gene expression models were compared. The groups with a poor outcome were those with a poor van`t Veer 70-gene profile, an activated wound response, a high recurrence score, and the basal-like, luminal B, and Her2+ and ERα-neg intrinsic subtypes. These data suggest that if a tumor is classified as basal-like, Her2+ and ERα-negative, or luminal B, then it is more likely to reside in the poor-prognosis groups of the 70-gene, wound-response, and recurrence score models [50]. Our differential abundance proteomics data are also compatible with those results.

Notably, several hemoglobin spots were all slightly more abundant in the ERα-pos tumor pool with marginal significance ($p > 0.05$: data not shown); their multiplicity suggesting that in addition to elevated lymphocyte levels [24], ERα-neg tumors may also have a lower density of blood vessels. Vascular Endothelial Growth Factor (VEGF) is a key mediator of tumor angiogenesis, including neovascularization in human breast cancer [51]. Garvin et al. suggest the operation of an estrogen-driven angiogenic switch, explained by the inhibition of soluble VEGFR-1 and stimulation of VEGF and VEGFR-2 tipping the angiogenic scale to favor angiogenesis, and possibly contributing to breast carcinoma progression [52]. Results obtained in a rodent experimental stroke model suggest that estrogen acting through its alpha receptor increases the expression of angiopoietin-1 mRNA and enhances capillary density in brain under basal conditions [53].

The most highly differential abundant protein in ERα-neg tumors relative to ERα-pos tumors in our study was Fibrinogen Gamma A Chain, followed by Fibrin. Fibrinogen is proteolytically cleaved under the influence of platelets to produce a fibrin clot during the process of blood coagulation after wounding. Cancer-related fibrin deposition and fibrinolysis characterizes many solid tumors, with cancer cells supplying many of the functions supplied by platelets in normal blood clotting [54]. The deposition of fibrinogen without subsequent conversion to fibrin in the tumor stroma is reportedly a hallmark of breast carcinoma [16].

Both fibrin and fibrin degradation products have been shown to promote angiogenesis, consistent with the hypothesis that fibrin-rich extracellular matrices may promote tumor stroma formation by mechanisms that are comparable with wound repair [55]. Additionally there is also substantial experimental evidence pointing to a role for fibrin(ogen) in tumor dissemination and metastasis [56]. The biology associated with variability in extracellular matrix of the different tumor ERα-pos and ERα-neg types revealed here deserves further examination.

We also detected that XTP3-Transactivated Protein A is significantly more abundant in ERα-neg tumors. This protein is a member of a recently identified superfamily of all-alpha NTP pyrophosphohydrolases, and is known to be overexpressed in embryonic and cancer cells [57].

Candidate PGRMC1 interacting proteins

It is reasonable to speculate that differences in the phosphorylation status of PGRMC1 can affect the proteins with which it interacts, and thereby affect cellular biology. The cancer relevance of PGRMC1 interactions with the proteins Insig-1 and SCAP which regulate the mevalonate pathway have been discussed above and reviewed [20]. Interaction of PGRMC1 with the protein PAIRBP1 may be involved in mediating an anti-apoptotic action of progesterone [58, 59]. Interestingly, PAIRBP1/CGI-55 was shown to interact not only with a cAMP-responsive element of the mRNA of a serpin inhibitor of extracellular proteases (for review: [20]), but also with the histone deacetlyase CHD3/Mi-2 [60]. This in turn is a component of the Nucleosome Remodeling and Deacetylase (NuRD) complex that is involved in chromatin remodeling that plays a prominent role in orchestrating events in breast cancer progression and metastasis involving epigenetic locus control by steroid receptors including the ERα in breast cancer [61, 62].

Concerning VEGF induction, vascularization, and possible PGRMC1 interaction partners, we note that migration of the vascular growth cone during angiogenesis is directed by the Netrin/DCC system [63]. The nematode homologs of these proteins are Unc-6/Unc-40 respectively. Netrin/Unc-6 is a lamin-like extracellular ligand for the DCC/Unc-40 receptor. Vem-1, the nematode homolog of PGRMC1, forms a protein complex with Unc-40 and the two genes were functionally linked by genetics [64]. Therefore PGRMC1 presumably interacts with the mammalian Unc-40 homologs Neogenin and/or DCC. The Netrin system is highly relevant to cancer because DCC and Neogenin are dependence receptors which have been argued to induce Caspase-directed apoptosis in the absence of Netrin [20, 65]. Intriguingly, a subpopulation of DCC resides in intracellular vesicles which can be relocalized to the plasma membrane [66], and PGRMC1 possesses a number of tyrosine-based (ITAM/YXXΦ) motifs which implicate a role in membrane trafficking [20, 64]. It is conceivable that PGRMC1 regulates the cellular location of DCC and/or Neogenin, as suggested [64], in a phosphorylation- and/or ligand-dependent dependent fashion.

Netrin and Neogenin/DCC are present in transmembrane extracellular protein complexes containing integrins and cadherins that mediate cell-cell contacts and generate cytoplasmic contact growth inhibition and survival signals [67, 68]. The Netrin system also is involved in the directed migration and tissue invasion of mammary terminal end buds, which are proliferative substructures which constitute the invading edge of developing mammary duct glands [69]. The latter example involves related cells to those that cause breast cancer, and is potentially highly relevant to our present study result. Indeed Neogenin promotes Endothelial-Mesoderm Transition [70], which correlates with local tumor invasion and metastasis [71]. Future research should address what role if any these proposed interactions of PGRMC1 with the candidate interaction partners discussed here may play in breast cancer.

Supplementary Tables

Protein Name	Number of Spots	Experimental PI	Experimental MW	Genbank AccNo	PMF Score
Albumin	19 spots	5.8	73000	gi|23307793	87
		5.3	71000	gi|6013427	54
		5.3	67000		72
		5.3	66000		58
		5.4	83000		75
		5.4	72000		93
		5.4	72000		96
		5.4	62000		85
		5.4	60000		70
		5.5	72000		112
		5.5	71000		158
		5.5	70000		86
		5.6	71000		136
		5.6	71000		144
		5.6	50000		63
		5.7	104000		94
		5.7	71000		88
		5.7	71000		90
		5.7	71000		128
ATP synthase	2 spots	6.7	55000	gi|24660110	83
		6.9	57000		169
Carbonic Ahydrase II	2 Spots	6.5	27000	gi|1633065	78
		6.7	26000	gi|999651	74
Cyclophilin A	3 Spots	6.8	16000	gi|1633054	87
		7.4	15000		81
		7.0	16000		85
Fibrinogen beta	5 spots	6.1	59000	gi|399492	79
		6.1	57000		85
		6.8	55000		66
		7.1	57000		88
		5.6	40000	gi|2781208	70
HSP27	2 spots	5.4	26000	gi|662841	123
		5.5	26000		125
Keratin 7	3 spots	5.2	55000	gi|30089956	97
		5.2	54000		112
		5.3	56000		273
Keratin 8	4 spots	5.3	56000	gi|39645331	154
		5.1	49000	gi|4504919	102
		5.2	49000		215
		5.4	55000		419
Keratin 9	4 spots	5.1	67000	gi|435476	217
		5.1	66000		141
		5.1	16000		132
		5.1	12000	gi|4557705	72
Keratin 19	4 spots	5.0	43000	gi|34783124	407
		5.0	42000		395
		5.0	42000		424
		4.9	41000		204
Alpha 1 antitrypsin	14 spots	4.5	60000	gi|1942629	126
		4.8	62000		103
		4.8	54000		97
		4.8	52000		71
		4.8	51000		93
		4.8	50000		80
		4.9	63000		186
		4.9	50000		97
		5.0	61000		232

Protein Name	Number of Spots	Experimental PI	Experimental MW	Genbank AccNo	PMF Score
		5.0	60000		254
		5.0	49000		149
		5.0	61000		130
		5.1	60000		133
		4.9	50000		66
Translation elongation factor delta	2 spots	4.9	35000	gi|25453472	93
		5.0	35000		70
PGRMC1	3 spots	4.6	22000	gi|5729875	95
		4.55	22000		110
		4.5	22000		107
Transferrin receptor	2 spots	6.0	80000	gi|37747855	99
		6.2	81000	gi|4557871	73
Transgelin	2 spots	7.8	22000	gi|4507359	95
		8.5	21000		137
Vimentin	3 spots	4.8	47000	gi|4507895	336
		5.0	56000		531
		4.9	45000		150

Supplementary Table S1.

Protein spots that contained multiple identifications of individual proteins as gene products. The protein name and number of spots are indicated in the column headings. Approximate estimates for the experimentally observed isoelectric point (PI) and molecular weight (MW) are given for each spot, as are Genbank accession numbers and PMF scores, the nomenclature conventions for which follow Figure 3.

Experimental variable designation		Description	Tumor status	Lymph node status	Grade	ER status	PR status	Her2/neu status	Age of patient
Figure 8 B	i-iii	invasive ductulo-lobular adenocarcinoma	2	0	2	6	9	1	52
	iv	invasive lobular adenocarcinoma	X	0	2-3	8	2	0	75
	v	invasive lobular adenocarcinoma	1	1	2	0	6	0	62
	vi	invasive ductulo-lobular adenocarcinoma	4	X	2	8	2	3	68
	vii	invasive ductal adenocarcinoma	2	1	2-3	0	0	3	69
	viii	invasive ductal adenocarcinoma	2	1	2	8	2	0	61
	ix	invasive ductal adenocarcinoma with ductal in situ component	1	X	2-3	12	6	0	72
	x	ductal in situ carcinoma	X	X	X	12	0	3	59
	xi	invasive ductal adenocarcinoma with ductal in situ component	1	0	2	12	12	0	73
	xii	invasive ductal adenocarcinoma	1	2	2	0	0	0	59
Figure 8 C	i-vii	multifocal invasive ductulo-lobular adenocarcinoma with carcinoma in situ	1	x	2	12	12	1	40

Supplementary Table S2.

Clinical patient data for the tumours in Figure 8B and 8C. Classifications are according to Table 1 in the main manuscript.
